# Nystatin Regulates Axonal Extension and Regeneration by Modifying the Levels of Nitric Oxide

**DOI:** 10.3389/fnmol.2020.00056

**Published:** 2020-04-03

**Authors:** Cristina Roselló-Busquets, Marc Hernaiz-Llorens, Eduardo Soriano, Ramon Martínez-Mármol

**Affiliations:** ^1^Department of Cell Biology, Physiology and Immunology, Faculty of Biology and Institute of Neurosciences, University of Barcelona, Barcelona, Spain; ^2^Centro de Investigación Biomédica en Red Sobre Enfermedades Neurodegenerativas (CIBERNED), ISCIII, Madrid, Spain; ^3^Institució Catalana de Recerca i Estudis Avançats (ICREA), Barcelona, Spain; ^4^Clem Jones Centre for Ageing Dementia Research (CJCADR), Queensland Brain Institute (QBI), University of Queensland, St Lucia Campus, Brisbane, QLD, Australia

**Keywords:** nystatin, axon growth, nitric oxide synthase, growth cone, axon regrowth post-axotomy

## Abstract

Nystatin is a pharmacological agent commonly used for the treatment of oral, mucosal and cutaneous fungal infections. Nystatin has also been extensively applied to study the cellular function of cholesterol-enriched structures because of its ability to bind and extract cholesterol from mammalian membranes. In neurons, cholesterol level is tightly regulated, being essential for synapse and dendrite formation, and axonal guidance. However, the action of Nystatin on axon regeneration has been poorly evaluated. Here, we examine the effect of Nystatin on primary cultures of hippocampal neurons, showing how acute dose (minutes) of Nystatin increases the area of growth cones, and chronic treatment (days) enhances axon length, axon branching, and axon regeneration post-axotomy. We describe two alternative signaling pathways responsible for the observed effects and activated at different concentrations of Nystatin. At elevated concentrations, Nystatin promotes growth cone expansion through phosphorylation of Akt; whereas, at low concentrations, Nystatin enhances axon length and regrowth by increasing nitric oxide levels. Together, our findings indicate new signaling pathways of Nystatin and propose this compound as a novel regulator of axon regeneration.

## Introduction

Mammalian adult Central Nervous System (CNS) differs from embryonic CNS and Peripheral Nervous System (PNS) by their inherent ability to regenerate lesioned tissues. After axotomy, the first regeneration step requires the formation of a functional growth cone. Unfortunately, the adult CNS has a reduced capacity to form new growth cones due, to the existence of intrinsic factors (Ertürk et al., 2007) and the presence of growth-inhibitory molecules (Tan et al., 2005; Li et al., 2013). After axotomy, organized sequential steps are required to form new and functional growth cones. The first of which consists of the influx of calcium, which increases exocytosis to fuse additional membrane to form a sealing patch to repair the ablated axon (Bradke et al., 2012; Blanquie and Bradke, 2018; Curcio and Bradke, 2018). Following this initial membrane addition, microtubule and actin cytoskeleton is reorganized, multiple signaling cascades are activated and the new membrane is transported to the tip of the growing axon (Bradke et al., 2012; He and Jin, 2016; Curcio and Bradke, 2018). A tight control of the actin cytoskeleton is crucial for the formation and functionality of the new growth cone. Regulation of actin requires the initiation of the phosphatidylinositol-3-kinase (PI3K)/Akt signaling cascade (Henle et al., 2011; Kakumoto and Nakata, 2013; Berry et al., 2016; Curcio and Bradke, 2018; Jin et al., 2018). Akt phosphorylation induces the activation of nitric oxide synthase (NOS), whose function is associated with actin reorganization and cell survival (Michell et al., 1999; Van Wagenen and Rehder, 2001; Welshhans and Rehder, 2005; Cooke et al., 2013; Sild et al., 2016). NOS produces nitric oxide (NO), a gaseous molecule involved in neurotransmission, neuronal growth and filopodia formation (Van Wagenen and Rehder, 2001; Welshhans and Rehder, 2005; Tojima et al., 2009; Forstermann and Sessa, 2012). NO is also associated with axon regeneration in insect neurons (Stern and Bicker, 2008) and the snail *Helisoma trivolvis* (Cooke et al., 2013). NO cannot be stored in cells, so its effects depend on the* de novo* synthesis by NOS activity. From the three types of NOS, neural NOS (nNOS) is synthesized in CNS and PNS neurons and its activity is regulated by intracellular calcium levels. The NO downstream signaling pathway involves the activation of protein kinase G (PKG) and actin-associated proteins such as the Enabled/vasodilator-stimulated phosphoprotein (Ena/VASP), resulting in a strong reorganization of the actin cytoskeleton (Zhou and Zhu, 2009; Forstermann and Sessa, 2012; Cossenza et al., 2014).

Nystatin is a drug commonly used as an antifungal agent because of its ability to destabilize fungal membranes by binding and extracting ergosterol, causing changes in cell permeability and, eventually, cell lysis (Bolard, 1986; Coutinho et al., 2004). Nystatin can also bind to cholesterol and extract this lipid from the membranes of mammalian cells. As a consequence, Nystatin has been widely used to disrupt and study the cellular function of lipid rafts. Lipid rafts are membrane microdomains enriched in cholesterol and sphingolipids, that facilitate the compartmentalization of signaling proteins, working as platforms for spatial and temporal regulation of the cytoskeleton, membrane anchoring, and cell adhesion, controlling the motility of growth cones (Guirland and Zheng, 2007), and the regenerative properties of lesioned axons (Tassew et al., 2014; Roselló-Busquets et al., 2019). The extended clinical use of Nystatin, together with its ability to affect the organization of lipid rafts, makes it an ideal candidate to explore its function as a possible therapeutic agent for the treatment of spinal cord lesions.

Here, we performed an *in vitro* evaluation of the Nystatin-induced axonal regenerative properties, analyzing the effect of various concentrations and incubation times of this compound in hippocampal neurons. The study of the downstream signaling proteins responsible for the observed effects of Nystatin suggested that Nystatin differentially activates Akt phosphorylation and NO production in a concentration-dependent manner. We propose Nystatin as a novel neuronal pharmacological regulator of Akt and nNOS activity that modifies growth cone dynamics and promotes axonal regeneration post-axotomy.

## Materials and Methods

### Reagents and Antibodies

The following antibodies were used: Mouse Anti-IIIβ-tubulin (MMS-435P, Covance), Rabbit mAb Anti-P-Akt (Ser473; #4060, Cell Signaling), Goat Anti-Akt (C-20; sc-1618, Santa Cruz), Donkey anti-Mouse IgG (H + L) Highly Cross-Adsorbed Secondary Antibody, Alexa Fluor 488 (A-21202, Thermo Fisher), Swine Anti-Rabbit Immunoglobulins/HRP (P0217, Dako), Rabbit Anti-Goat Immunoglobulins/HRP (P0449, Dako).

The following drugs and reagents were used: Poly-D-Lysine (P7280, Sigma), rat tail collagen Type I, Rat Tail (354236, Corning), Nystatin dihydrate (N4014, Sigma), DMSO (D5879, Sigma), Methyl-β-cyclodextrin (C4555, Sigma), Phalloidin—TRITC (P1951, Sigma), NG-Monomethyl-L-arginine, monoacetate salt (L-NMMA; ab120137, Abcam), diamino-fluorescein Diacetate (DAF-FM DA; D-23844, Molecular Probes), CellTracker™ RedCMTPX Dye (C34552, Thermo Fisher), Complete Protease Inhibitor Cocktail Tablets (11697498001, Roche), MK-2206-2HCl (A10003, AdooQ Bioscience).

### Neuronal Cultures

Hippocampal and forebrain primary cell cultures and explants were obtained from E16-E17 (embryonic day 16–17) mice embryos. Pregnant CD1 dams were sacrificed by cervical dislocation and the fetuses were collected and decapitated. Brain tissues were maintained during the dissection procedure constantly submerged in ice-cold 0.3% glucose-phosphate-buffered saline (PBS) solution. For primary cell cultures, hippocampi or forebrains were isolated and trypsinized for 6 min at 37°C. Trypsin was neutralized with FBS, the tissues were incubated with DNase I for 10 min at 37°C, and then they were mechanically dissociated by gentle trituration. The neurons were centrifuged at 800 rpm for 5 min, resuspended and plated in culture glasses pre-coated with 0.5 mg/ml poly-D-lysine. The composition of the neuronal culture medium was Neurobasal (w/o L-glutamine, w/ Phenol Red; 21103-049, GIBCO), 1% penicillin/streptomycin (15140-122, GIBCO), 1% Glutamine (25030-024, GIBCO) and 2% B27 (17504-044, GIBCO). Explants were obtained from dissected hippocampi, plated in 15.6 mm dishes pre-coated culture glasses with 0.5 mg/ml poly-D-lysine (P7280, Sigma) and 0.03 mg/ml collagen (354236, Corning) with neuronal culture medium. Explants were cultured for 3 DIV (3 days *in vitro*) in the experiments of axon extension, or were cultured for 7 or 14 DIV in the experiments of axon regeneration. After 7 or 14 days, axotomy was performed using a hypodermic needle (302200, BD Microlance) to cut the axons close to the explant body (Finn et al., 2000). Axotomized explants were collected using a pipette and moved to a new dish, where they will be immersed in a collagen matrix. When collagen was coagulated, culture media was applied (Lumsden and Davies, 1986) and explants were kept in culture for 3 more days with the corresponding treatments.

### Drug Treatments

For acute treatment experiments on dissociated forebrain and hippocampal neurons to measure growth cone size, filopodia density, Akt phosphorylation, and NO formation, cells were incubated with Nystatin at the following concentrations: 2.5 μM, 10 μM or 25 μM during 30 min. DMSO was used as vehicle control condition, applied to match the same volume of Nystatin used.

For chronic treatment experiments on hippocampal explants and dissociated neurons to measure axonal extension and regeneration, the samples were incubated with 2.5 μM Nystatin or DMSO for 3 days. Nystatin was added to the culture medium immediately after plating dissected neurons (experiments of axon extension) or after axotomy (experiments of axon regeneration).

### Nitric Oxide Experiments

Primary hippocampal neurons were obtained as described above. After 3 DIV, neurons were pre-incubated with 100 μM L-NMMA or control medium for 1 h at 37°C. Immediately after, the pre-incubation medium was removed and neuronal NO was labeled by incubating with 5 μM DAF-FM supplemented with 100 μM L-NMMA or control medium for 30 additional minutes at 37°C. After three washes with Neurobasal, neurons were further incubated with 10 μM CellTracker™ RedCMTPX Dye in addition to 2.5 μM Nystatin or 0.5 μM MβCD supplemented with 100 μM L-NMMA or control medium for 30 min at 37°C. CellTracker was used as a marker of the surface of the neurons.

Hippocampal explants were cultured with 100 μM L-NMMA or control medium for 2 h immediately before axotomy was performed. Explants were then returned to their culture medium for three additional days in the presence of 2.5 μM Nystatin or DMSO, supplemented with or without L-NMMA.

### Akt Inhibition Experiments

Primary hippocampal neurons were obtained as described above. After 3 DIV, neurons were pre-incubated with 2 μM MK-2206-2HCl or control medium for 4 h at 37°C. Immediately after, the pre-incubation medium was removed and neurons were incubated with 2.5, 10, 25 μM Nystatin or control medium supplemented with or without 2 μM MK-2206-2HCl for 30 min at 37°C.

### Immunocytochemistry

Hippocampal dissociated cultures were fixed with 4% PFA in PBS for 10 min at room temperature (RT), permeabilized with 0.1% PBS-Triton for 10 min. To detect the actin cytoskeleton, neurons were stained with a solution of 1 μg/ml phalloidin-TRITC in PBS for 30 min, rinsed with PBS and mounted in Mowiol. To detect P-Akt, Akt or tubulin, neurons were incubated with a blocking solution, 10% normal horse serum (NHS) in TBS, for 2 h and with primary antibody diluted in blocking solution for 2 h. Then, neurons were washed and incubated with secondary antibody in blocking solution for 1 h rinsed with TPBS and mounted in Mowiol. To detect cholesterol, neurons were fixed with a solution of 0.12 mM sucrose in 4% PFA in PBS for 15 min at RT. Then, neurons were stained with a freshly prepared solution of 0.05mg/ml filipin in PBS for 90 min, rinsed with PBS, fixed again with 0.12 mM sucrose in 4% PFA for 20 min and mounted in Mowiol (Gu et al., 1997; Feng et al., 2003).

Explants were fixed with a solution of 4% PFA in PBS or 30 min at RT. Then, the explants were rinsed with PBS and permeabilized with a solution of 0.5% Triton X-100 in PBS for 30 min. Explants were then incubated with blocking solution, NHS 10% in PBS, for 2 h. After blocking, explants were incubated overnight at 4°C with the primary antibody diluted in blocking solution. Explants were then washed three times with PBS and incubated with the respective secondary antibodies diluted in blocking solution for 2 h at RT. Finally, explants were washed three times in PBS and mounted in Mowiol.

### Immunoblotting

Forebrain neurons were dissected and cultured in 35 mm diameter dishes during 3 DIV. On the third day, neurons were treated with control medium (containing DMSO) or Nystatin medium (at different concentrations) for 30 min. After each respective treatment, neurons were placed on ice and lysed with ice-cold lysis buffer supplemented with a protease inhibitor cocktail (11697498001, Roche), 10 mM NaF, 1 mM Na_3_VO_4_ and 10 mM Na_2_H_2_P_2_O_7_. Cell lysates were diluted with loading buffer and boiled for 5 min. Samples were separated by electrophoresis using an 8% polyacrylamide gel. Proteins were transferred to nitrocellulose membranes (10600002, GE Healthcare Life Sciences) and incubated with different primary antibody and secondary antibodies. Bands were quantified using GelPro Analyzer software (version 3.1, Media Cybernetics).

### Image Acquisition

Images from explants, growth cones and filopodia were acquired using an epifluorescence microscope (Eclipse Nikon E1000) under a 5× and 10× objective (for explants) or a 60× oil-immersion objective (for growth cones and filopodia). A confocal microscope (Leica TCS SP5) was used to acquire a z-stack of images (every 0.5 μm) from DAF-FM and P-Akt intensity with 63x oil-immersion objective.

### Image Analysis and Quantifications

Each experiment contains a mixed culture of neurons isolated from more than three embryos. All experiments were repeated three independent times (independent dissections). or the analysis of growth cone area and filopodia density, actin staining through phalloidin was used to identify growth cones and axonal filopodia. Filopodia were manually counted as actin-enriched protrusions formed in discrete segments along the axon. Proximal (<50 μm) and distant (>50 μm) axonal regions were randomly selected for quantifications. The growth cone compartment was outlined based on a differential staining for actin in the growth cone concerning the axon compartment. An intensity threshold mask was created using ImageJ (Schneider et al., 2012) and the growth cone perimeter was selected using the wand tool (similar results were obtained by manual selection of growth cone perimeter). In the DAF-FM experiments, the intensity was measured (mean gray value × area) inside the cell body (maximum of Z projections) using ImageJ. The measurements were normalized to the signal intensity obtained in control conditions, to avoid basal fluorescence. Between 20–30 images were acquired for each condition. In P-Akt images, the intensity was quantified inside growth cones and in the cell body, and was normalized to the control condition. In the explant growth and regeneration experiments, the signal from dapi staining (nuclei) was subtracted from each explant and considered as the beginning of the axons. In 3 DIV and 7 DIV explants, the length of eight lines drawn from the beginning of the axons until their most distal part was measured. In 14 DIV explants, the 10 longest axons were selected and their length from the beginning of the axons until their tip was measured. For each explant, 8 (in 7 DIV) or 10 (in 14 DIV) axon measurements were obtained and averaged.

### Statistical Analysis

All the data shown in the graphs represent the mean ± SEM. The number of neurons and explants used in each experiment is specified in the corresponding figure legend. Data was analyzed using GraphPad software. Normal distribution was evaluated by applying the D’Agostino and Pearson normality test. Two-tailed, unpaired Student’s *t*-test was used to compare two conditions, and one-way ANOVA with Tukey *post hoc* test was used when the experiment had more than two conditions. Significance is considered when *p-value < 0.05, **p < 0.01, ***p < 0.001.

## Results

### Different Doses of Nystatin Affect Growth Cone Size

**Figure 1:**
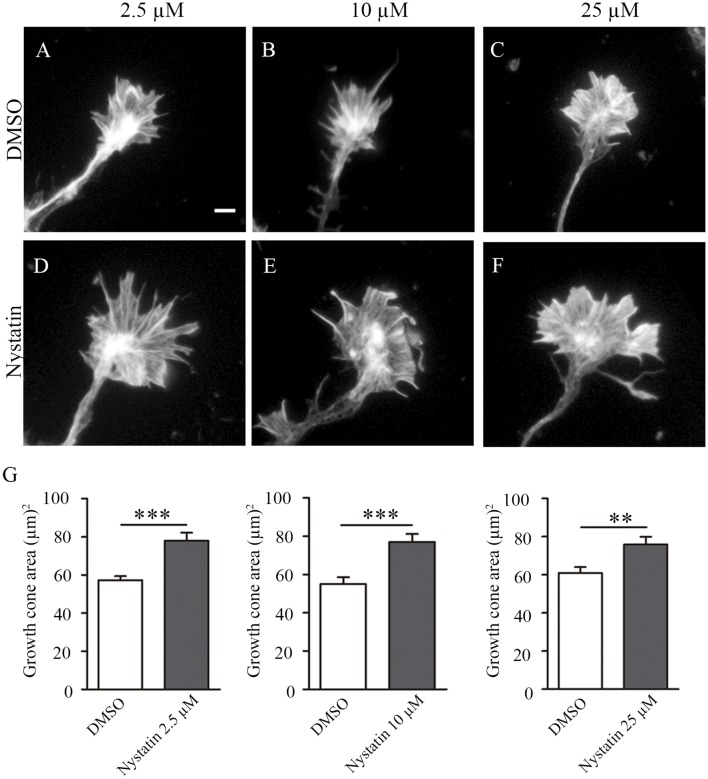
Acute incubation with different concentrations of Nystatin increases the growth cone size of hippocampal neurons. Representative images of growth cones from hippocampal neurons cultured during 3 DIV and incubated with control medium (DMSO; **A–C**) or Nystatin at 2.5 μM **(D)**, 10 μM **(E)** and 25 μM **(F)** for 30 min. Growth cone area quantification for each treatment **(G)**. Neuronal actin was stained incubating cells with phalloidin-TRITC (1 μg/ml) for 30 min. Data shows mean ± SEM. *n* = 80–120 neurons in each condition. Two-tailed, unpaired Student’s *t*-test was performed. **p < 0.01, ***p < 0.001. Scale bar 5 μm.

Growth cones play a key role in axon regeneration and membrane cholesterol levels can modify growth cone dynamics. Nystatin is a polyene antifungal agent widely used in experimental research to alter cholesterol levels and modify the function of lipid rafts. We recently proposed a reduction of cholesterol levels as a possible strategy to promote axon regrowth after sciatic nerve lesion (Roselló-Busquets et al., 2019). However, the potential applicability of Nystatin remains unexplored. To answer this question, we first analyzed the effect of different concentrations of Nystatin on the growth cone area in hippocampal neurons (Figure 1). We cultured mice hippocampal neurons for 3 days *in vitro* (3 DIV) and treated them acutely with different widely used concentrations of Nystatin, from high doses that can remove membrane cholesterol (25 μM) to low doses that do not affect cholesterol levels (2.5 μM; Johnson et al., 1998; Koide et al., 2009; Kim et al., 2013). All three concentrations tested, 2.5 μM (Figures 1A,D), 10 μM (Figures 1B,E) and 25 μM (Figures 1C,F) increased growth cone area (Figure 1G).

### Different Doses of Nystatin Affect Differentially Akt Phosphorylation

**Figure 2:**
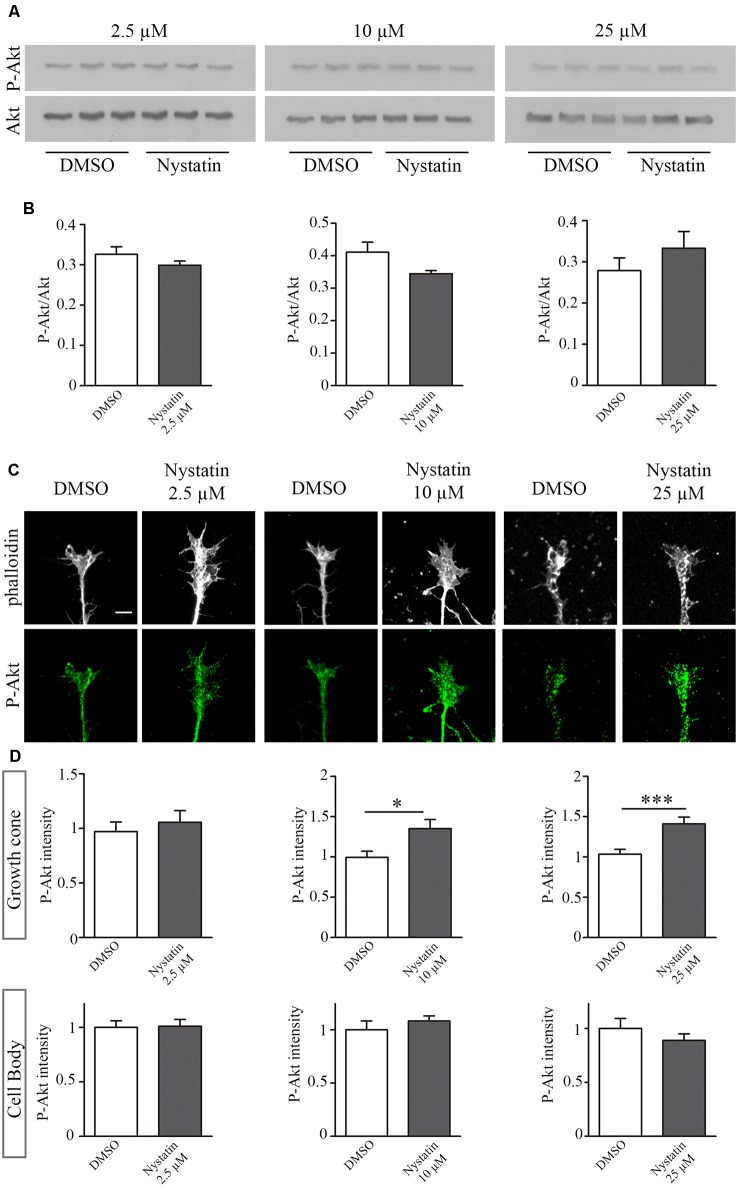
Dose-specific Nystatin effect to Akt phosphorylation. Western blots from E16 cortex primary cell cultures, cultured during 3 DIV and incubated with control medium (DMSO) or Nystatin 2.5 μM, 10 μM or 25 μM for 30 min. P-Akt and Akt were detected **(A)**. The ratio of P-Akt/Akt was quantified with Gelpro software. *n* = 3 neuronal extracts in each condition. Two-tailed, unpaired Student’s *t*-test was performed **(B)**. Representative images of hippocampal growth cones treated with Nystatin at the doses described above, stained with phalloidin and labeled against P-Akt **(C)**. Quantification of the relative P-Akt intensity in the growth cones and neuronal cell bodies **(D)**. Data shows mean ± SEM. *n* = 30–50 neurons in each condition. Two-tailed, unpaired Student’s *t*-test was performed. *p < 0.05, ***p < 0.001. Scale bar 5 μm.

The results from Figure 1 suggest a cholesterol-independent effect of Nystatin in controlling growth cone dynamics. To find out the mechanism through which Nystatin is increasing growth cone size, we studied whether Akt phosphorylation is affected under our three Nystatin concentrations tested. Western Blot analysis of P-Akt/Akt levels from primary cultured forebrain neurons treated with 2.5 μM, 10 μM and 25 μM of Nystatin (Figure 2A) revealed no significant differences, but only a tendency in increasing Akt phosphorylation under the highest dose tested (Figure 2B). However, the evaluation of specific P-Akt levels in growth cones (Figure 2C) and cell bodies by immunocytochemistry assay showed that only the highest doses of Nystatin (10 μM and 25 μM) significantly increase Akt phosphorylation locally inside growth cones (Figure 2D).

### High Doses of Nystatin Increase Growth Cone Area Through Akt Phosphorylation

**Figure 3:**
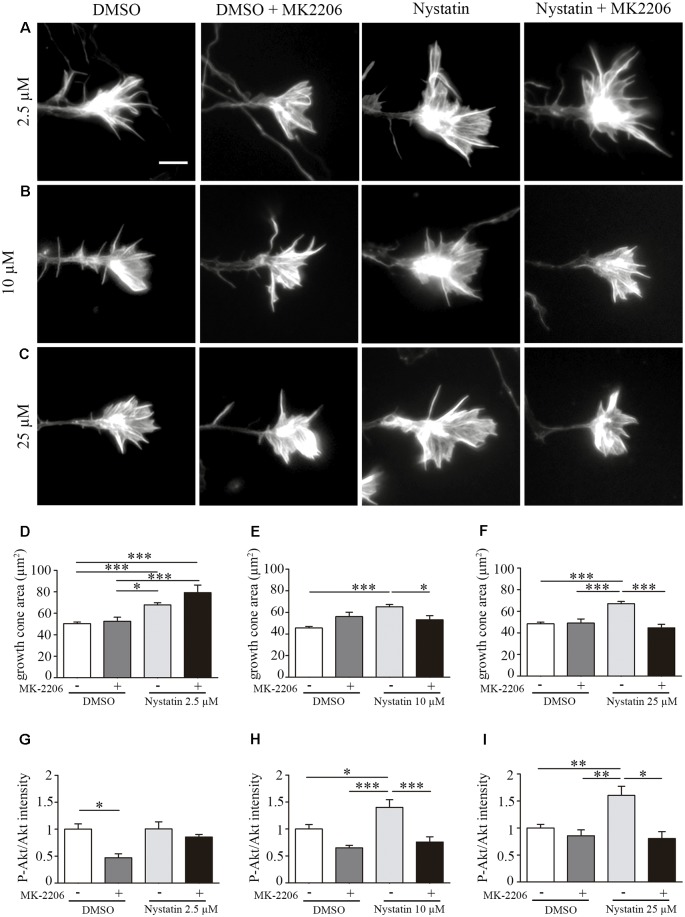
Effect on growth cone size by high concentrations of Nystatin requires Akt phosphorylation. Representative images of growth cones from hippocampal neurons cultured during 3 DIV and incubated with Nystatin at 2.5 μM **(A)**, 10 μM **(B)** and 25 μM **(C)** for 30 min in the presence or not of Akt inhibitor MK-2206. Growth cone area quantification for each treatment **(D–F)**. Neuronal actin was stained incubating cells with phalloidin-TRITC (1 μg/ml) for 30 min. Immunocytochemistry quantification of the ratio of P-Akt/Akt in growth cones for each treatment **(G–I)**. Data shows mean ± SEM. *n* = 20–30 neurons for Akt intensity and 150–200 neurons for growth cone area in each condition. One-way ANOVA, Tukey’s multiple comparison test; *p < 0.05, **p < 0.01, ***p < 0.001. Scale bar 5 μm.

To analyze whether Nystatin Akt phosphorylation is required for the observed effects of Nystatin on growth cones, we used the Akt inhibitor MK-2206. Whereas lowest concentration of Nystatin (2.5 μM) increases growth cone area independently of Akt inhibition (Figures 3A,D), the effect induced by highest doses (10 μM and 25 μM) depends on Akt activity (Figures 3B,C,E,F). These results are consistent with the phosphorylation of Akt in growth cones. Immunocytochemistry analysis of P-Akt/Akt levels within growth cones revealed that those concentrations of Nystatin that increased Akt phosphorylation also affected growth cone dynamics in an Akt-dependent manner (Figures 3G–I).

### Nystatin Increases Axon Growth in Hippocampal Explants

**Figure 4:**
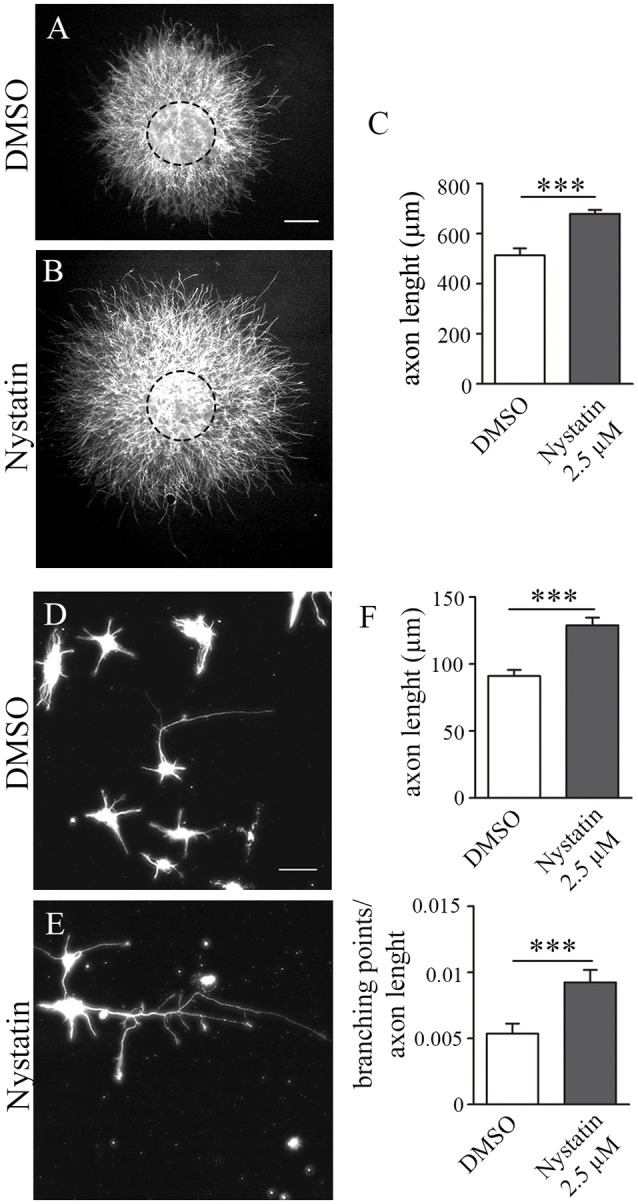
Chronic treatment of Nystatin increases axon length and branching in hippocampal neurons. Representative images of hippocampal explants cultured inside a collagen matrix **(A,B)** or dissociated neurons **(D,E)**, cultured in the presence of DMSO control media **(A,D)** or 2.5 μM Nystatin **(B,E)** for 3 DIV. Axon length quantification from explants **(C)** or dissociated neurons (**F**, upper graph). Quantification of the density of branching points in the axon (**F**, lower graph). Neuronal class III β-tubulin was immunoassayed to identify axons and measure their length. Data shows mean ± SEM. *n* = 15–20 explants, 200 neurons in each condition. Two-tailed, unpaired Student’s *t*-test was performed ***p < 0.001. Scale bar 250 μm **(A,B)**, 50 μm **(D,E)**.

We then wanted to evaluate whether chronic treatments of Nystatin promote axonal growth in hippocampal neurons. To avoid possible toxicity effects of longer exposure to Nystatin, we used the lowest concentration tested (2.5 μM) which was sufficient to promote a significant increase in growth cone size (Figure 4). Hippocampal explants were cultured inside a collagen matrix with control media (Figure 4A) or with 2.5 μM Nystatin-containing media (Figure 4B) for 3 days. The length of explant-protruding axons was analyzed, revealing that chronic exposure to a low concentration of Nystatin promotes axon growth (Figure 4C). We then used primary cell cultures of hippocampal neurons (Figures 4D,E) to study the chronic effect of 2.5 μM Nystatin on axonal growth and branching. After 3 days of incubation with Nystatin, we found increases on both axon length and axonal branching (Figure 4F).

### Nystatin Increases the Growth Cone Area and Filopodia Density Through NO Production

**Figure 5:**
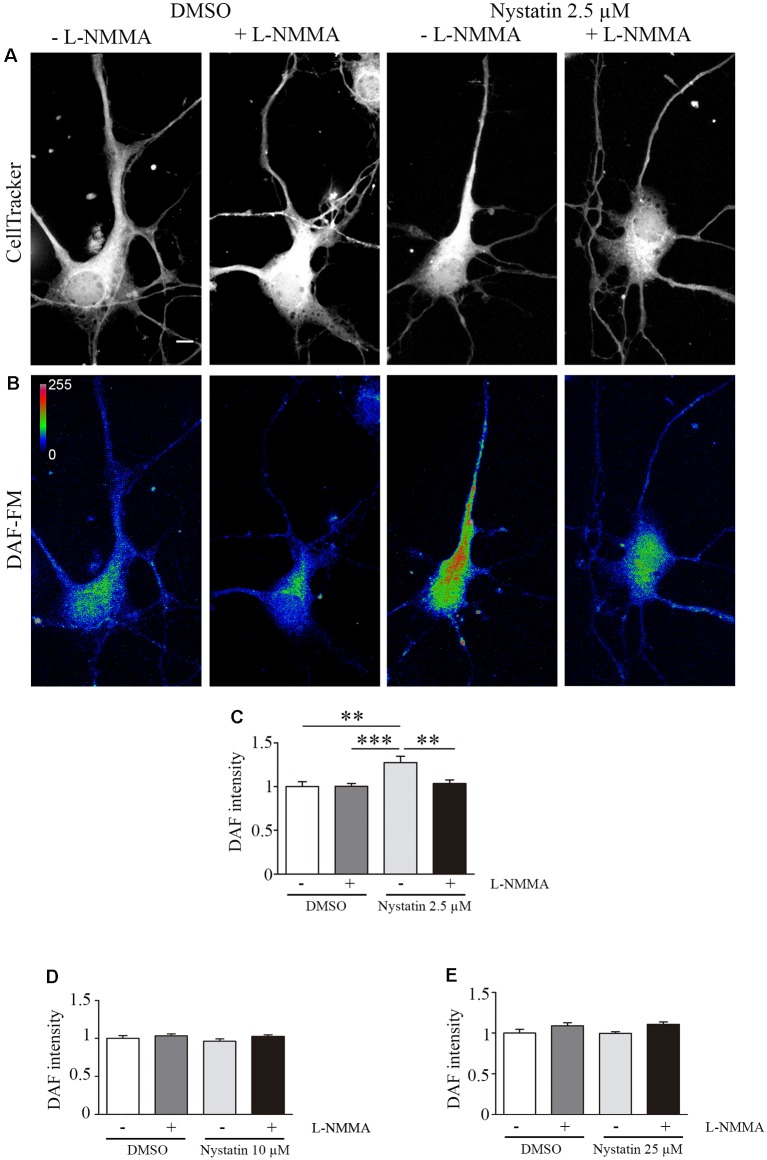
Nystatin increases nitric oxide production in hippocampal neurons. Representative images of hippocampal neurons stained with CellTracker™ Dye **(A)** and DAF-FM **(B)** to detect nitric oxide production under the presence of DMSO control conditions (± the NOS inhibitor L-NMMA) or 2.5 μM Nystatin (± the NOS inhibitor L-NMMA) incubated during 30 min. Images in **(B)** are shown in a pseudo-color scale where magenta color indicates high levels of NO and blue color indicates low levels of NO. DAF-FM intensity was quantified in each condition and presented relative to the DMSO control condition **(C)**. Similarly, DAF-FM intensity was also quantified for neurons treated with 10 μM Nystatin **(D)** or 25 μM Nystatin **(E)** and presented relative to DMSO control condition. Data shows mean ± SEM. For each condition, *n* = 20–30 neurons were used for DAF intensity, *n* = 150–200 growth cones (one per neuron) for growth cone area, and *n* = 40–60 axons for filopodia density. One-way ANOVA, Tukey’s multiple comparison test; **p < 0.01, ***p < 0.001. Scale bar 5 μm.

**Figure 6:**
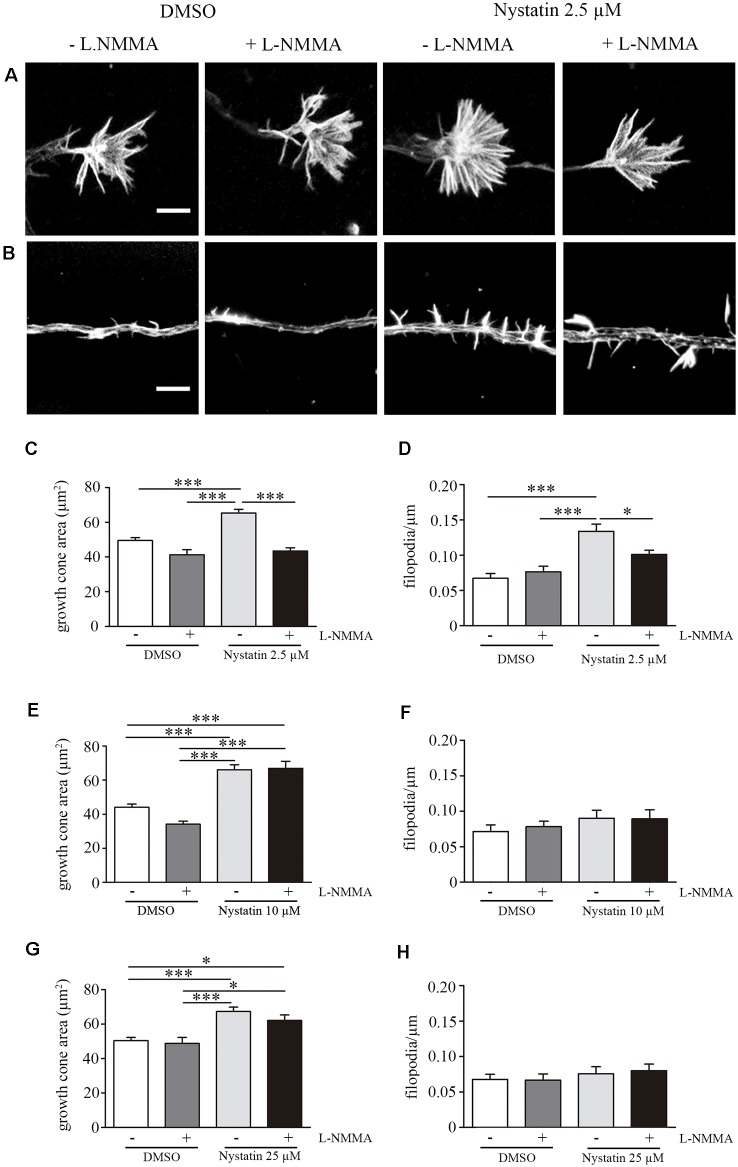
Acute treatment of Nystatin increases growth cone and filopodia density trough nitric oxide production. Representative images of growth cones **(A)** and filopodia **(B)** treated during 30 min with DMSO (± L-NMMA) or 2.5 μM Nystatin (± L-NMMA). Quantification of growth cone area **(C)** and filopodia density **(D)** for each treatment under DMSO or 2.5 μM Nystatin. Similarly, growth cone area **(E,G)** and filopodia density **(F,H)** were also quantified for neurons treated with 10 μM Nystatin **(E,F)** or 25 μM Nystatin **(G,H)**. Neuronal actin was used to identify growth cone morphology and axon filopodia. Actin was stained by incubating cells with phalloidin-TRITC (1 μg/ml) for 30 min. Data shows mean ± SEM. *n* = 20–30 neurons in each condition. One-way ANOVA’s multiple comparison test; *p < 0.05, ***p < 0.001. Scale bar 5 μm.

Our results suggest a cholesterol and Akt phosphorylation independent effect on growth cone dynamics by low concentrations of Nystatin in acute (min) and chronic (days) treatments. NO is a gaseous second messenger that participates in actin cytoskeleton remodeling, filopodia formation (Welshhans and Rehder, 2005) and growth cone guidance (Tojima et al., 2009). To evaluate whether the observed effects of low concentrations of Nystatin on growth cones depend on NO production, we incubated hippocampal neurons with a NOS inhibitor (L-NMMA) and detected the formation of NO using Diamino-fluorescein Diacetate (DAF-FM DA), a cell-permeable reagent used to quantify low concentrations of NO in solution. DAF-FM DA remains non-fluorescent until it is hydrolyzed to DAF-FM by intracellular esterases, allowing its reaction with NO to form a fluorescent benzotriazole. DAF fluorescent intensity was quantified, revealing increased levels of NO upon low-dose Nystatin treatment (Figures 5A–C). Importantly, the treatment of neurons with the highest concentrations of Nystatin (10 μM and 25 μM) did not affect NO production (Figures 5D,E). The effect of Nystatin in the growth cone area (Figure 6A) and filopodia density (Figure 6B) was prevented by NOS inhibition (Figures 6C,D), suggesting NO production as an alternative mechanism used by Nystatin to modulate axon dynamics at low concentrations. Consistent with the lack of action on NO production, the effect of highest doses of Nystatin (10 μM and 25 μM) on growth cone area and filopodia density is independent of NOS inhibition (Figures 6E–H). Methyl-beta-cyclodextrin (MβCD) is a compound that, similar to Nystatin, extracts cholesterol from cell membranes, increasing growth cone area and filopodia number (Roselló-Busquets et al., 2019). NO measurement using DAF-FM showed that MβCD treatment did not affect NO production (Supplementary Figure S1). These results suggest that the growth cone area and filopodia number can be modulated by decreasing cholesterol levels or by regulating NO production using low concentrations of Nystatin.

### Nystatin Increases Axon Regeneration in Immature and Differentiated Hippocampal Explants Through NO Production

**Figure 7:**
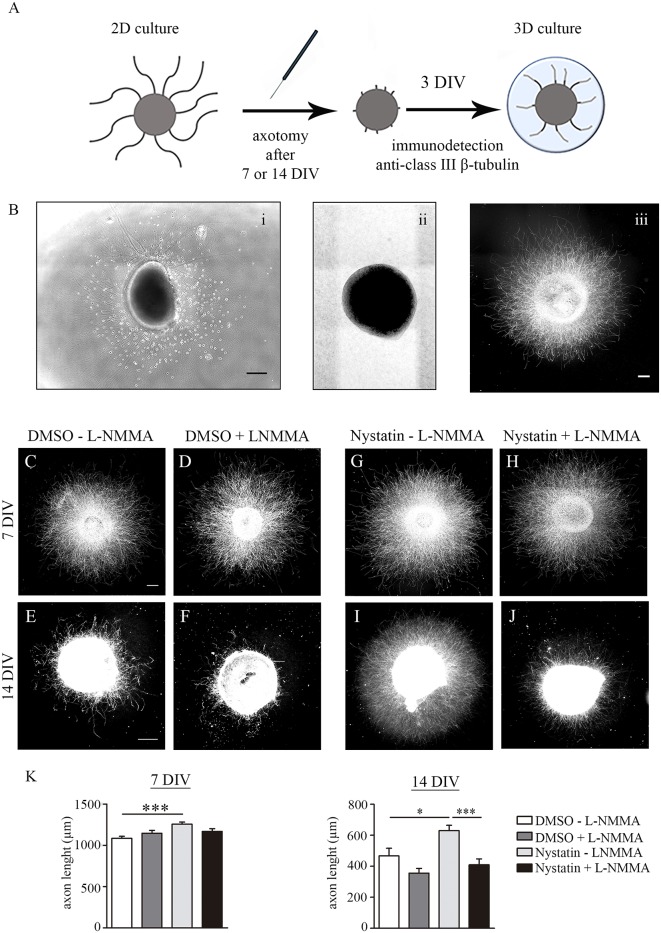
Chronic treatment of Nystatin increases axon regeneration through nitric oxide production. Scheme of axotomy procedure **(A)**. Representative bright-field images of explants before axotomy **(Bi)** and immediately after axotomy **(Bii)**. Representative immunofluorescence image of an explant 3 days after axotomy **(Biii)**. Scale bar 200 μm. Representative images of hippocampal explants axotomized after 7 DIV or 14 DIV and regrowth for three additional days inside a collagen matrix in control medium **(C–F)** or 2.5 μM Nystatin **(G–J)**, and without the NOS inhibitor L-NMMA **(C,E,G,I)** or with the NOS inhibitor L-NMMA **(D,F,H,J)**. Neuronal class III β-tubulin was immunoassayed to identify axons and measure their length. Quantification of explant axon length **(K)**. Data shows mean ± SEM. *n* = 25–30 explants in each condition. One-way ANOVA, Tukey’s multiple comparison test; *p < 0.05, ***p < 0.001. Scale bar 250 μm.

Previous results showed that Nystatin increases axon regeneration post-axotomy in primary cell cultures of hippocampal neurons (Roselló-Busquets et al., 2019). The ability of axons to regenerate is lost in adult CNS neurons (He and Jin, 2016; Curcio and Bradke, 2018; Fawcett, 2019). Primary CNS neuronal cultures lose their regenerative capacities during their *in vitro* differentiation (del Rio and Soriano, 2010). To study whether low concentrations of Nystatin promote axon regeneration post-axotomy and whether this effect is maintained in differentiated neurons, we performed axotomy experiments with hippocampal explants cultured during 7 and 14 DIV (Figures 7A,B). Then, axon explants were mechanically ablated and further regrowth for 3 DIV in the presence of 2.5 μM Nystatin supplemented with the NOS inhibitor L-NMMA (Figures 7C–J). Quantification of axon length revealed that Nystatin promotes regrowth of axons after axotomy regardless of the differentiation state of the neuronal cultures, and in a process that requires NO production (Figures 7C–K).

## Discussion

This study examines the mechanisms through which Nystatin, a drug used normally to treat fungal infections, enhances axonal growth and regeneration using differential dose-dependent mechanism. We demonstrate that only high concentrations of Nystatin increase the size of growth cones through Akt phosphorylation, whereas low concentrations exert the same effect by promoting NO production. The formation of NO is necessary for the chronic effect of Nystatin on modulating axon dynamics and promoting regeneration post-axotomy.

Nystatin can activate Akt phosphorylation in macrophages (Kim et al., 2013), but to date, the effect has completely been unexplored in neurons. The combination of Western Blot, Akt inhibitor, and immunocytochemistry analysis revealed a local growth cone increase of Akt phosphorylation. This effect is specifically localized in growth cones, explaining why it was not detected by Western Blotting. Activation of the PI3K/Akt pathway in growth cones produces an increase of the exocytosis (Laurino et al., 2005), a process necessary for membrane expansion and axon growth (Pfenninger, 2009; Cotrufo et al., 2011). Although low concentrations of Nystatin also increased the growth cone area, they did not enhance Akt phosphorylation. High concentrations of Nystatin require Akt phosphorylation to induce their effect on growth cone dynamics. It has been previously described that low doses of Nystatin increases NO levels in macrophage-like cell lines (Koide et al., 2009), but this effect had not been previously described in neurons. Our results are in agreement and suggest an alternative mechanism where Nystatin only at low concentrations controls growth cone dynamics by modifying NO levels. These results suggest that depending on its concentration, Nystatin could be acting through different pathways. High doses of Nystatin also remove membrane cholesterol from cell membranes; however, alteration of cholesterol levels using an elevated concentration of Nystatin does not affect NO production.

There is a discrepancy in the literature about the benefits of NO increments on neurites and growth cones. NO is necessary for neurite growth, axon guidance and filopodia length (Van Wagenen and Rehder, 2001; Welshhans and Rehder, 2005; Cooke et al., 2013; Sild et al., 2016). However, NO has also been associated with a growth cone collapse (Cossenza et al., 2014; Redondo et al., 2015). This discrepancy could be explained by the fact that NO effects depend on its concentration and the multiple possible interactions with other molecules. While low to moderate increment of NO levels is beneficial for cell survival, a high increment of NO concentration is associated with cell death (Cossenza et al., 2014). By using NOS inhibitors we find that the increment of NO-induced by only by low concentrations of Nystatin is required to promote axon regeneration after axotomy.

Disruption of cell membrane permeability has also been associated with the production of reactive oxygen species (ROS). It has been described that an increment in ROS levels is necessary to initiate axon regeneration after sciatic nerve and spinal cord injury (Hervera et al., 2018), and to regulate F-actin dynamics in the growth cones and neurite outgrowth through Rac1 (Munnamalai and Suter, 2009). NO degradation results in the formation of ROS. Mutations in Cu/Zn superoxide dismutase (SOD1), an enzyme that converts superoxide radicals to molecular oxygen and hydrogen peroxide, are associated with increments of growth cone area, filopodia density, axonal growth and branching in adult motor neurons due to the accumulation of ROS (Osking et al., 2019). The formation of ROS as secondary sub-products of NO formation could be involved in the observed phenotype after Nystatin treatments.

In conclusion, here we demonstrate that Nystatin could activate two different pathways in neurons, PI3K/Akt and nNOS/NO, and that nNOS activity is necessary for axonal regeneration when Nystatin is applied chronically at 2.5 μM. With this study, we propose that Nystatin, a drug currently used as an antifungal agent and to extract cholesterol from the cell membranes, might have an alternative effect improving axon growth and regeneration. Our findings suggest Nystatin as an interesting candidate molecule to be tested in neuronal re-growth and repair.

## Data Availability Statement

The raw data supporting the conclusions of this article will be made available by the authors, without undue reservation.

## Ethics Statement

The animal study was reviewed, approved and was carried out in accordance with the recommendations of the European Communities Council Directive 2010/63/EU. The protocol was approved by the Ethics Committee on Animal Experimentation of the Universitat de Barcelona.

## Author Contributions

RM-M, CR-B, and ES designed the research. CR-B and MH-L performed the experiments. RM-M, CR-B, and MH-L analyzed the data. RM-M, CR-B, MH-L, and ES made the figures and wrote the manuscript. RM-M and ES supervised the study.

## Conflict of Interest

The authors declare that the research was conducted in the absence of any commercial or financial relationships that could be construed as a potential conflict of interest.

## Supplementary Material

FIGURE S1 Cholesterol disruption with MβCD does not affect nitric oxide production. Representative images of hippocampal neurons stained with CellTracker™ Dye **(A)** and DAF-FM **(B)** to detect nitric oxide production under control conditions (± the NOS inhibitor L-NMMA) or 0.5 μM MβCD (± the NOS inhibitor L-NMMA). Images in **(B)** are shown in a pseudo-color scale where magenta color indicates high levels of NO and blue color indicates low levels of NO. DAF-FM intensity was quantified in each condition and presented relative to the DMSO control condition **(C)**. Data shows mean ± SEM. *n* = 20–30 neurons in each condition. One-way ANOVA, Tukey’s multiple comparison test. Scale bar 5 μm.
