# Supplementary figures and images for: Nystatin Regulates Axonal Extension and Regeneration by Modifying the Levels of Nitric Oxide
Source: Front Mol Neurosci. 2020 Apr 3;13:56. doi: 10.3389/fnmol.2020.00056 (PMC7146717; doi:10.3389/fnmol.2020.00056)

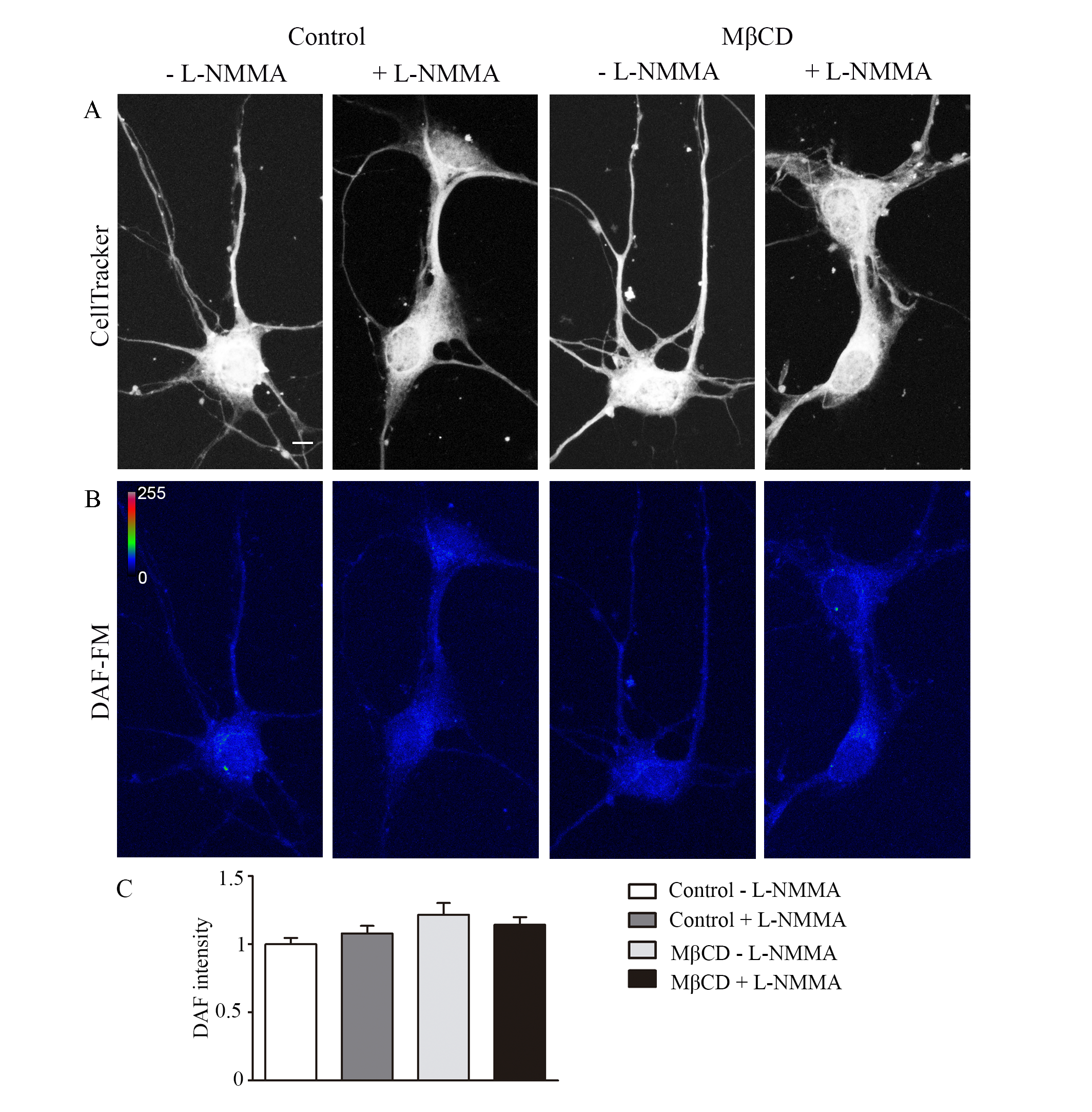

Supplement: FIGURE S1 — Cholesterol disruption with MβCD does not affect nitric oxide production. Representative images of hippocampal neurons stained with CellTracker™ Dye (A) and DAF-FM (B) to detect nitric oxide production under control conditions (± the NOS inhibitor L-NMMA) or 0.5 μM MβCD (± the NOS inhibitor L-NMMA). Images in (B) are shown in a pseudo-color scale where magenta color indicates high levels of NO and blue color indicates low levels of NO. DAF-FM intensity was quantified in each condition and presented relative to the DMSO control condition (C). Data shows mean ± SEM. n = 20–30 neurons in each condition. One-way ANOVA, Tukey’s multiple comparison test. Scale bar 5 μm. [file Image_1.TIF]
